# Flexible fixation of syndesmotic diastasis using the assembled bolt-tightrope system

**DOI:** 10.1186/1757-7241-21-71

**Published:** 2013-09-22

**Authors:** Guohui Xu, Wei Chen, Qi Zhang, Juan Wang, Yanling Su, Yingze Zhang

**Affiliations:** 1 Department of Orthopaedic Surgery, Third Hospital of Hebei Medical University, Shijiazhuang, Hebei 050051, P.R. China

**Keywords:** Syndesmotic diastasis, Tightrope, Syndesmotic bolt, Assembled bolt-tightrope system, Flexible fixation

## Abstract

**Background:**

Syndesmotic diastasis is a common injury. Syndesmotic bolt and tightrope are two of the commonly used methods for the fixation of syndesmotic diastasis. Syndesmotic bolt can be used to reduce and maintain the syndesmosis. However, it cannot permit the normal range of motion of distal tibiofibular joint, especially the rotation of the fibula. Tightrope technique can be used to provide flexible fixation of the syndesmosis. However, it lacks the ability of reducing the syndesmotic diastasis. To combine the advantages of both syndemostic bolt and tightrope techniques and simultaneously avoid the potential disadvantages of both techniques, we designed the assembled bolt-tightrope system (ABTS). The purpose of this study was to evaluate the primary effectiveness of ABTS in treating syndesmotic diastasis.

**Methods:**

From October 2010 to June 2011, patients with syndesmotic diastasis met the inclusion criteria were enrolled into this study and treated with ABTS. Patients were followed up at 2, 6 weeks and 6, 12 months after operation. The functional outcomes were assessed according to the American Orthopedic Foot and Ankle Society (AOFAS) scores at 12 months follow-up. Patients’ satisfaction was evaluated based upon short form-12 (SF-12) health survey questionnaire. The anteroposterior radiographs of the injured ankles were taken, and the medial clear space (MCS), tibiofibular overlap (TFOL), and tibiofibular clear space (TFCS) were measured. All hardwares were routinely removed at 12-month postoperatively. Follow-ups continued. The functional and radiographic assessments were done again at the latest follow-up.

**Results:**

Twelve patients were enrolled into this study, including 8 males and 4 females with a mean age of 39.5 years (range, 26 to 56 years). All patients also sustained ankle fractures. At 12 months follow-up, the mean AOFAS score was 95.4 (range, 85 to 100), and all patients were satisfied with the functional recoveries. The radiographic MCS, TFOL, and TFCS were within the normal range in all patients. After hardware removal, follow-up continued. At the latest follow-up (28 months on average, (range, 25 to 33 months) from internal fixation), the mean AOFAS score was 96.3 (range, 85 to 100), without significant difference with those assessed at 12 months after fixation operations. No syndesmotic diastasis reoccurred based upon the latest radiographic assessment.

**Conclusions:**

ABTS can be used to reduce the syndesmotic diastasis and provide flexible fixation in a minimally invasive fashion. It seems to be an effective alternative technique to treat syndesmotic diastasis.

## Background

Syndesmotic diastasis was a common injury [1]. The key to treat syndesmotic diastasis is to achieve anatomical reduction and effective fixation of the distal tibiofibular joint, which permits syndesmotic ligament healing and restores the stability of ankle joint. If treated unproperly, sequelae such as latent diastasis, chronic instability, chronic pain, osteochondral lesions, or arthritic changes may develop [2]. The optimal method of syndesmotic fixation remains an ongoing-debate topic. Several fixation implants have been reported, including metal cortical screws, bioabsorbable screws, syndesmotic bolts and tightrope [3-10]. Metal cortical screw fixation is recommended by the AO organization, being the most commonly used treatment method. However, complications such as screw loosening or screw breakage are not rare [11]. The syndesmotic bolt, which can reduce and effectively maintain the syndesmosis, has been used for many years [12,13]. A modified syndesmotic bolt has recently been reported, which is more flexible than metal cortical screw fixation, permitting some degree of micromovement [14]. However, it cannot permit the normal range of motion of distal tibiofibular joint, especially the rotation of the fibula [14]. Syndesmotic tightrope and even more flexible fixator have been recently introduced [8,10,15-18]. Flexible fixation of the syndesmosis can be achieved using tightrope in a minimally invasive manner, and patients can start physical exercises earlier. Tightrope gets its popularization in treating syndesmotic diastasis. However, tightrope may become loose and syndesmotic diastasis may reoccur in a long term [19]. In addition, it lacks the ability of reducing syndesmotic diastasis [20,21]. Therefore, we designed the assembled bolt-tightrope system (ABTS) to combine the advantages of both the syndesmotic bolt and tightrope. We hypothesized that ABTS can effectively reduce the syndesmotic diastasis and also provide flexible fixation. The purpose of this study was to evaluate the primary clinical and radiographic outcomes of syndesmotic diastasis treated with ABTS.

## Methods

A prospective clinical study was designed to evaluate the effectiveness of ABTS in treating distal tibiofibular syndesmotic diastasis from October 2010 to June 2011. The inclusion criterion was that patients sustained distal tibiofibular syndesmotic diastasis with or without ankle fractures. Syndesmotic diastasis was defined as tibiofibular clear space (TFCS) more than 6.0 mm on the anteroposterior or mortise radiographs, tibiofibular overlap (TFOL) less than 6.0 mm on the anteroposterior radiograph or less than 1.0 mm on the mortise radiographs [22], or medial clear space (MCS) more than superior clear space or 5.0 mm on the anteroposterior radiographs [23]. The exclusion criteria included open ankle fractures or multiple trauma in the ipsilateral lower extremities, diabetes, neuropathic arthropathy, dementia and other disease which made patients unable to comply with instructions. The study was approved by the Institutional Review Board of the Third Hospital of Hebei Medical University, and each patient signed the informed consent form.

### Structure of ABTS

**Figure 1:**
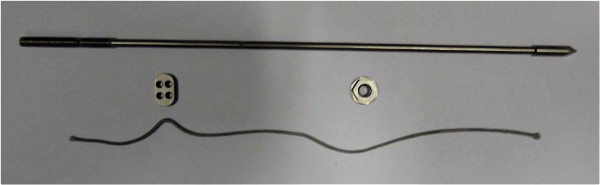
Four parts of ABTS, pre-cut nail, nut, button and the 2–0 FiberWire.

The ABTS consists of four parts (Figure 1): a pre-cut bolt, a nut and a button (Naton Medical Group Ltd., China), and a 2–0 FiberWire rope (Arthrex, Inc., Naples, FL). Each bolt has a 3-blade trocar tip with a diameter of 4.0 mm, a smooth rod of 3.0 mm in diameter with a pre-cut groove in the middle part and a threaded end with a diameter of 3.5 mm. The tip was used to create a trans-tibiofibular tunnel. There is a second pre-cut groove and a hole in the threaded end. The pre-cut groove is created for easy breaking of the bolt and the hole is used to secure the fiberwire. The nut, which is screwed onto the threaded rod, is 3.5 mm in height with a round bottom surface with a diameter of 10 mm. The button is oblong in shape and has 4 holes to anchor the fiberwire.

### Operative technique and postoperative management

**Figure 2:**
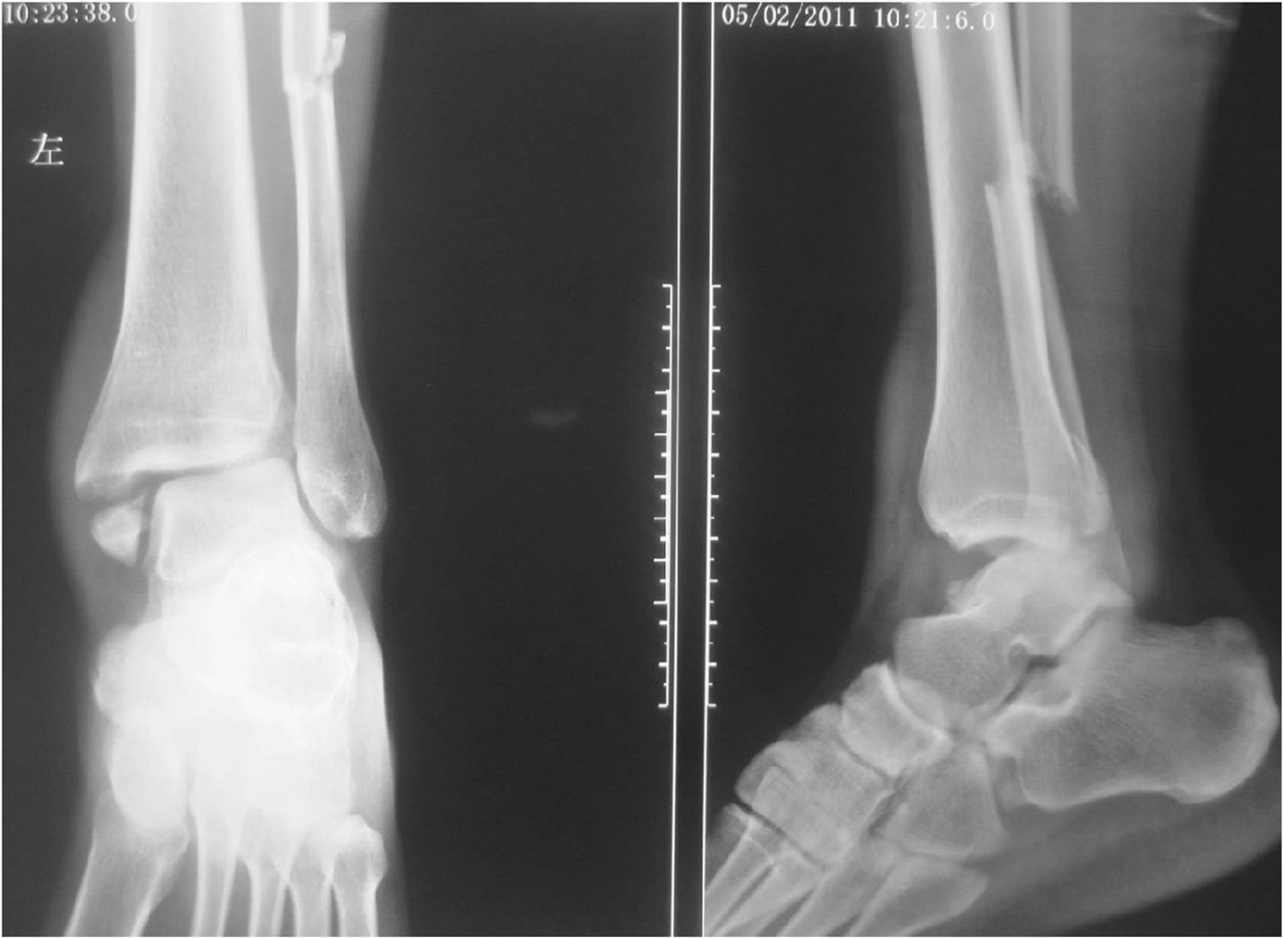
The pre-operation radiograph shows that the fracture type was 44C23 by AO classification combined with distal tibiofibular diastasis.

**Figure 3:**
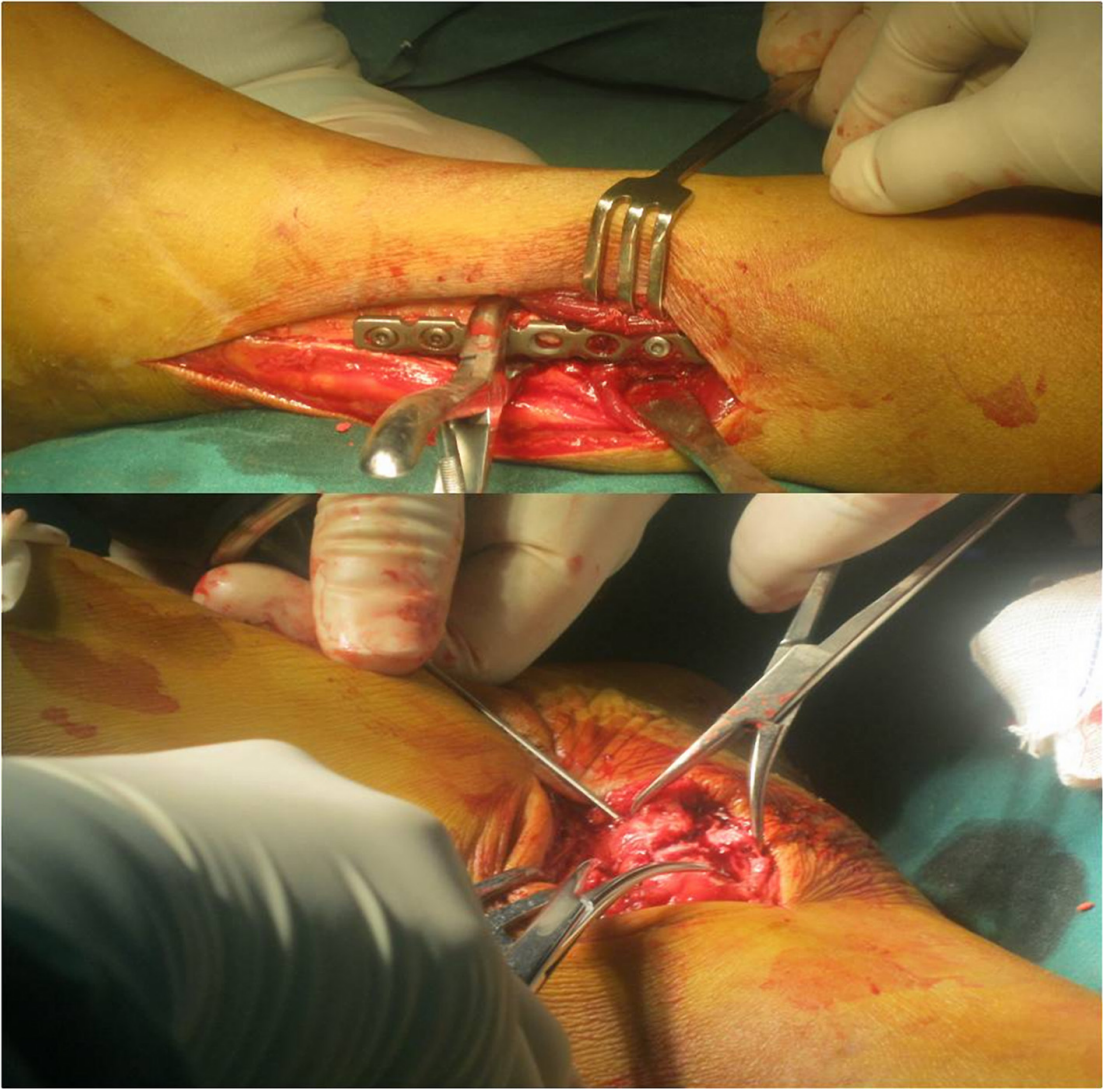
The fibular and medial malleolus fractures had been fixed.

All operations were performed by the senior author. The patients received spinal anesthesia and were positioned supine on the operative table. A thigh tourniquet was used. The associated fractures were firstly managed using AO technique (Figures 2 and 3). Some diastasis of the syndesmosis could be reduced spontaneously after open reduction and internal fixation of the ankle fractures. However, some diastasis remained after the management of ankle fractures, which should be reduced firstly with a reduction clamp or using ABTS itself.

**Figure 4:**
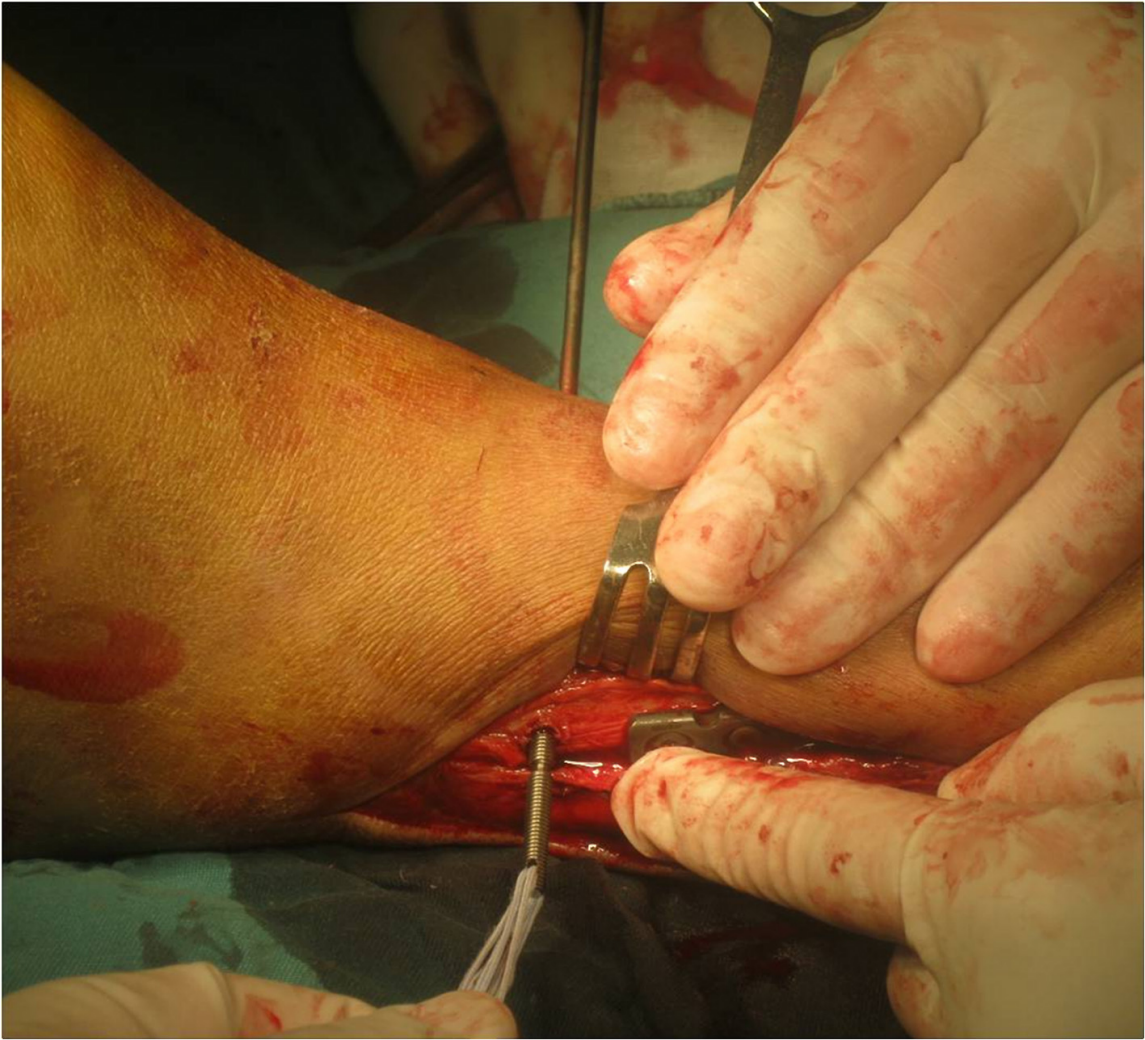
The tunnel had been created and the device was pulling from lateral to medial by hand.

The new device was inserted either directly through the fibula or through the hole of a fibular plate after internal fixation of fibular fractures. If it was inserted directly through the fibula, a 10.0 mm skin incision was made over the fibula, or the incision for the fixation of proximal fibula fracture was extended distally. A 4.0 mm tunnel was drilled under fluoroscopic guidance using the trocar tip of the bolt from fibula to tibia, which was 2–5 cm proximal and parallel to ankle joint line and angled approximately 30 degrees anteriorly (Figure 4). After the trocar tip penetrated the medial skin, a 10.0 mm skin incision was made over the penetrating point. Such an incision was unnecessary if any prior medial incision existed. The bolt was then pulled medially under fluoroscopic guidance to leave a proper length in the tunnel. Two strands of folded fiberwire were inserted through the bolt hole, so that there were four folded strands existing in the lateral side of the fibula after bolt inserted. Each folded strand was penetrated via one hole of the 4-hole oblong button.

**Figure 5:**
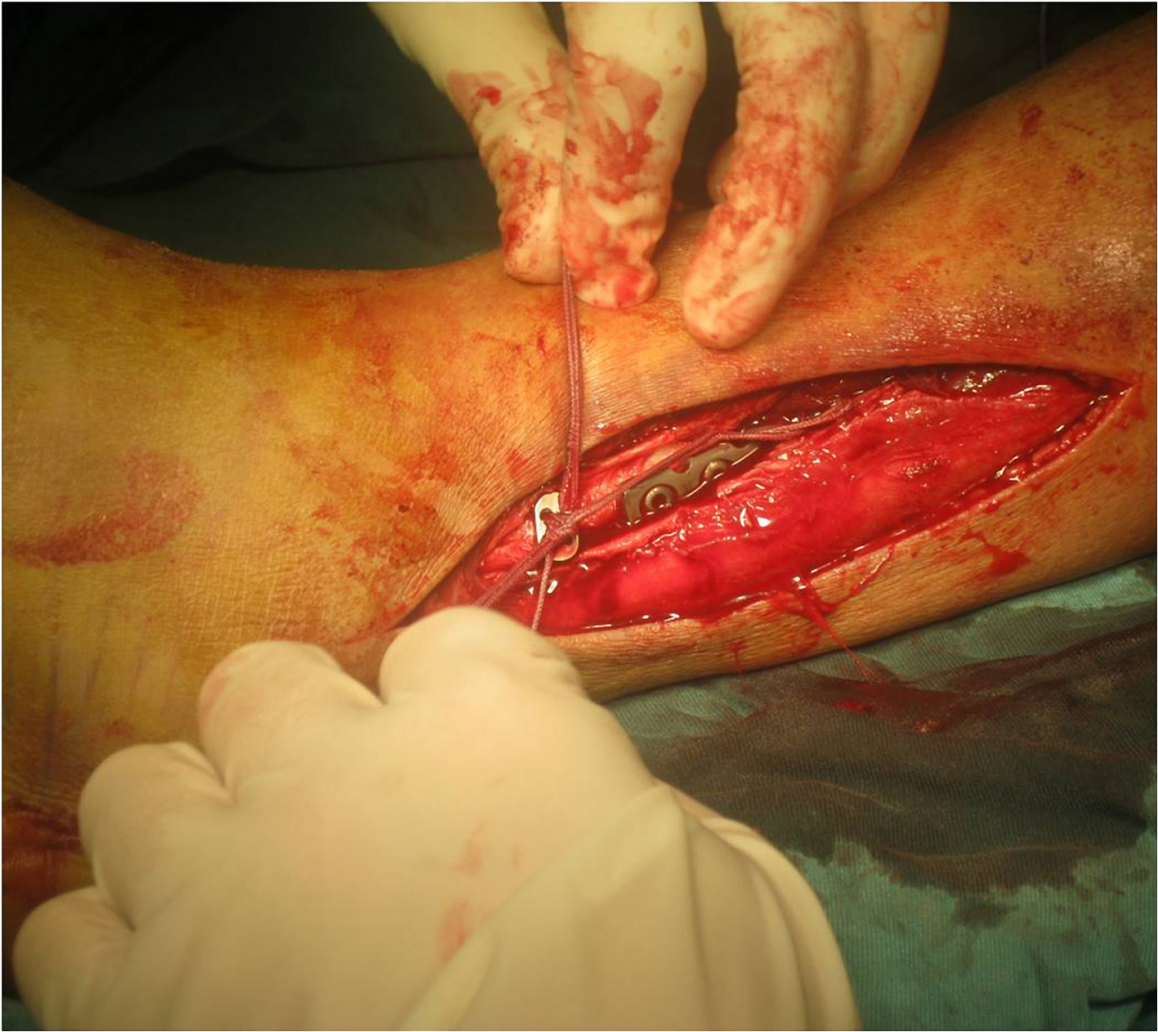
The nut was tightened.

**Figure 6:**
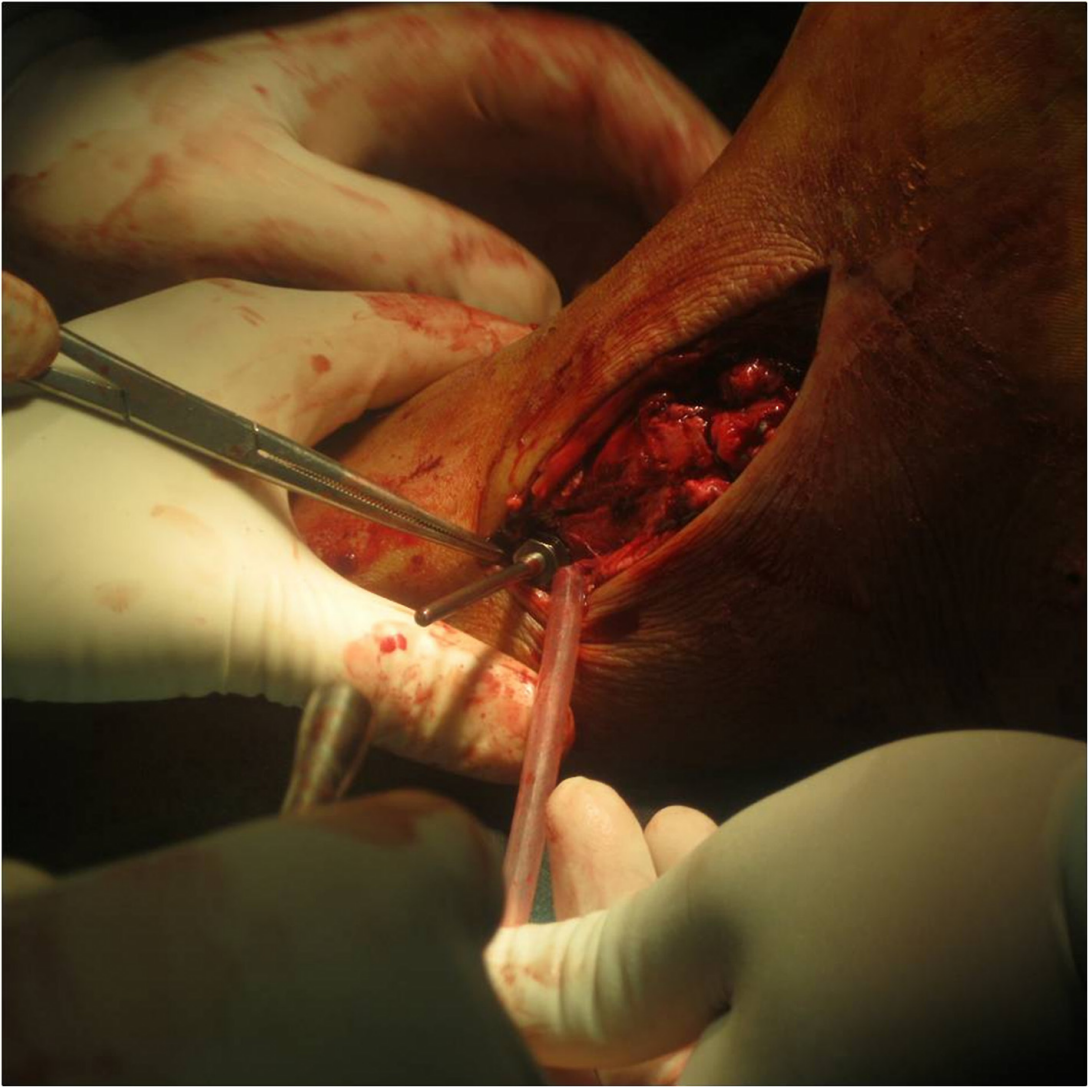
Knots were making after the nail was pulled to the proper position.

The smooth portion of the bolt was broken off at the first pre-cut groove to remove the trocar tip. The nut was screwed onto the threaded rod and rotated, by which enormous force can be generated to reduce the syndesmotic diastasis gradually (Figure 5). Finally, the remaining threaded bolt was broken off at the second pre-cut groove. Ideally, the tip of the retained bolt should be flush with the nut and beneath the skin after break off. There were some surgical tips for ideal selection and insertion of ABTS. If anatomical reduction of syndesmotic diastasis was achieved before ABTS insertion, the second pre-cut groove could be positioned 1 or 2 mm outside of the medial cortex of the tibia, so that the nut can be appropriately tightened. If the syndesmotic diastasis was reduced using ABTS, the second pre-cut groove should be withdrawn into the tunnel for a distance equal to the increased width of the tibiofibular clear space. Then the button was pushed onto the fibula and two knots were made tightly using the four folded strands with each having at least 3 half-hitches (Figure 6). The bolt was fastened gradually by the nut until the second pre-cut groove was exposed when anatomical reduction of the syndesmosis could be achieved.

After wound closure, the ankle was immobilized in the neutral position with a non-weight bearing below-knee cast for the first two weeks. If fracture fixation was stable and the wound healed well, the patient was placed in a below-knee walking cast or walker boot, allowing partial weight-bearing (50% body weight) at two weeks postoperatively. However, if the fracture fixation was unstable, the patient remained in a non-weight bearing cast for another four weeks. Full weight-bearing was allowed at six weeks postoperatively.

All patients were followed up at 2 weeks, 6 weeks, 6 months and 12 months postoperatively. At 12-month follow-up, ABTS was routinely removed and follow-up continued. Radiographic and functional outcomes were assessed at 12-month and the latest follow-up. Syndesmotic integrity was evaluated by measuring MCS, TFOL and TFCS on the radiographs of the affected ankles. The distance of ABTS from tibial plafond was also measured and recorded. The functional recoveries of the affected ankles were assessed by AOFAS ankle-hindfoot scores. Patients’ satisfaction was assessed based upon the short form-12 (SF-12) health survey questionnaire.

## Results

During the study period, twelve patients with syndesmotic diastasis were identified and treated with ABTS. All patients sustained ankle fractures of the ipsilateral lower extremities. The demographics of the patients, the mechanism of injury and the patterns of ankle fractures are summarized in Table 1. During operation, ankle fractures were firstly reduced and fixed with plates and screws. In five cases, the syndesmotic diastasis was reduced spontaneously after anatomical reduction and internal fixation of ankle fractures, and then fixed with ABTS. In the other seven cases, the diastasis remained and was reduced and fixed with ABTS. ABTS was inserted through a fibular plate hole in six cases and directly through the fibula in the other six cases. The postoperative course was uneventful and no major complications, such as loss of reduction, wound problems, implant loosening, or osteolysis, were reported.

**Table 1:** Patients’ demographics, mechanism of injury and fracture patterns

**Total number**	**12**
**Gender**	
**Male**	8
**Female**	4
**Age (mean, years)**	39.5
**Side**	
**Left**	5
**Right**	7
**Mechanism of injury**	
**Fall from height**	3
**Car accident**	5
**Hit by heavy**	2
**At home**	2
**AO classification**	
**44C12**	2
**44C13**	1
**44C21**	1
**44C22**	1
**44C23**	7

**Figure 7:**
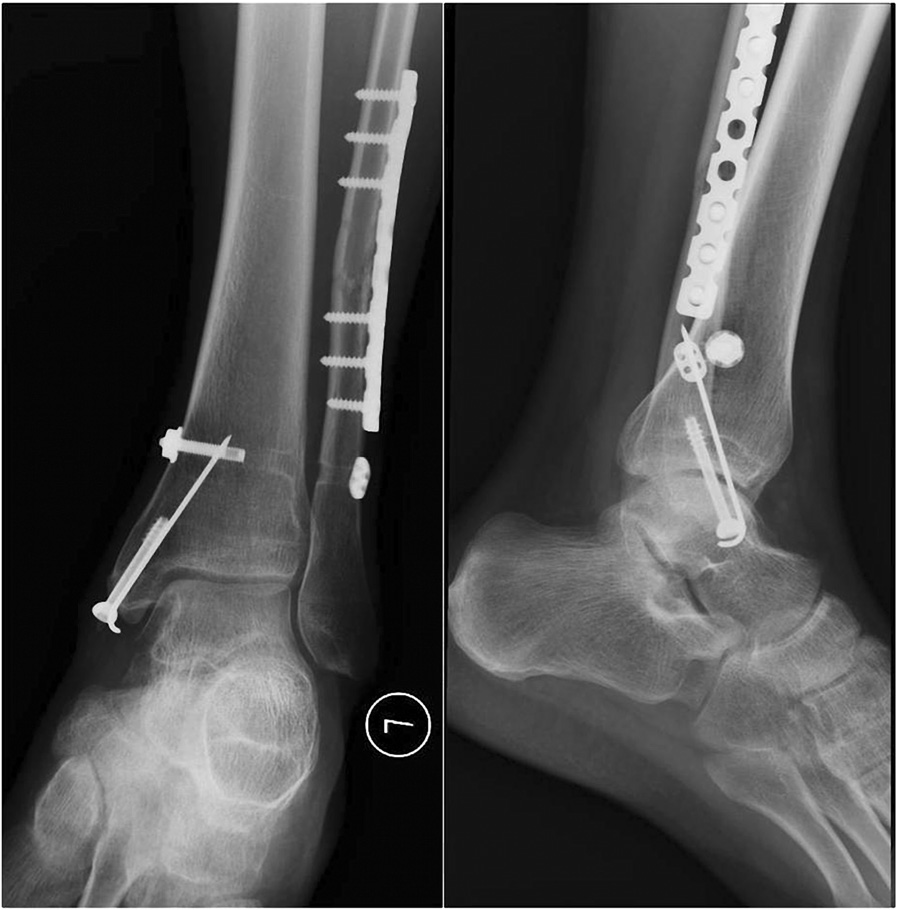
Mortise and lateral view of the ankle joint radiograph showed union of the fibular and medial malleolus fractures and a normal syndesmosis at 1 year follow-up after operation.

**Figure 8:**
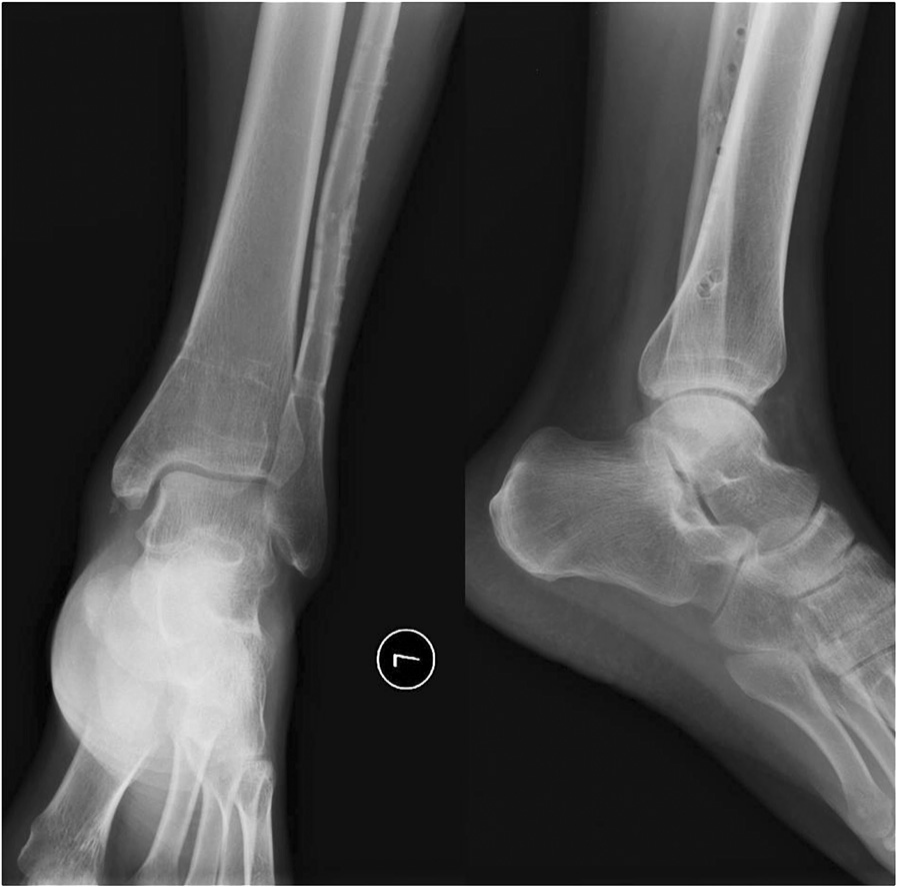
**The photograph was taken at 2 weeks after hardwares removal operation when patient returned to normal walking.** This photograph showed that all hardwares were removed and no syndesmotic diastasis reoccured.

The patients were followed up. The clinical and radiological evaluations (Figure 7) at 12-month are summarized in Table 2. All patients were satisfied with the outcomes. The hardware was routinely removed at 12-month follow-up. All patients were followed up for an average of 13.8 months (range, 12 to 17 months) after hardware removal (Table 2). At the latest follow-up, the mean AOFAS score was 96.3, without significant difference with those assessed at 12-month after fixation operations; the mean MHS and PHS were 57 and 50.3, respectively, without significant difference with those assessed at 12-month after fixation operations. No syndesmotic diastasis reoccurred (Figure 8). The mean value of MCS, TFOL and TFCS was 3.2 mm, 8.4 mm and 4.1 mm, respectively (Table 2). There were no significant differences between MCS, TFOL and TFCS at 12-month follow-up and those assessed at the latest follow-up, respectively (all *P* > 0.05).

**Table 2:** The clinical and radiological evaluations

**Time to full weight-bearing**	**6 weeks**
**Distance from tibial plafond**	30.5 mm (range 23.7-39.1)
**MCS**	
**Pre-op**	9.1 mm (range 6.8-13.9)
**Post-op at 12-month follow-up**	3.1 mm (range 2.4-4.1)
**At the latest follow-up**	3.2 mm (range 2.4-4.1)
**TFCS**	
**Pre-op**	9.8 mm (range 6.9-13.2)
**Post-op at 12-month follow-up**	4.1 mm (range 3–5)
**At the latest follow-up**	4.1 mm (range 3.1-5.1)
**TFOL**	
**Pre-op**	2.0 mm (range 0–5.8)
**Post-op at 12-month follow-up**	8.4 mm (range 7–9.3)
**At the latest follow-up**	8.4 mm (range 7–9.2)
**AOFAS score**	
**Post-op at 12-month follow-up**	95.4 (range 85–100)
**At the latest follow-up**	96.3 (range 85–100)
**SF-12**	
**12 months postoperative**	
**MHS**	56.4 (range 41–65)
**PHS**	49.9 (range 36–60)
**At the latest follow-up**	
**MHS**	57 (range 41–65)
**PHS**	50.3 (range 36–60)

*MCS* medial clear space, *TFCS* tibiofibular clear space, *TFOL* tibiofibular overlap, *AOFAS* American Orthopedic Foot and Ankle Society, *SF-12* Short Form-12 Health Survey questionnaire, *MHS* mental health summary, *PHS* physical health summary.

## Discussion

The syndesmotic bolt and tightrope are two useful techniques of treating syndesmotic diastasis [12,14,16-18,21]. The syndesmotic bolt can reduce the diastasis effectively. A modified syndesmotic bolt allows some degree of micro-movement of the tibiofibular joint, and seldom requires hardware removal [14]. However, it cannot permit full range of scale motion of the joint, especially the rotation of the fibula. Tightrope technique can achieve flexible fixation of the syndesmosis and permit full range of motion of the tibiofibular joint. Patients can start rehabilitation exercise at an early stage after operation. In addition, the tightrope does not require removal and there is no concern about hardware breakage [15]. However, tightrope technique lacks the reduction ability. The syndesmosis must be reduced first often with a large reduction clamp before fixation with tightrope [20,21]. Addtionally, there is some concern about its ability to maintain the reduction [8,16]. A recent study showed a significant increase in diastasis during external rotation force acting on the injured syndesmosis of cadaveric specimens fixed with tightrope, when compared with those fixed with a 4.5 mm cortical screw inserting across 4 cortices [24]. Another potential concern is that the medial button might be pulled into the metaphyseal cortex, leading to reduction failure [21].

To avoid the potential disadvantages of both syndesmotic bolt and tightrope techniques, we designed the assembled bolt-tightrope system (ABTS). ABTS technique is a minimally invasive and easy-to-perform procedure, which combines the advantages of syndesmotic bolt with those of tightrope technique. The medial “bolt” part of ABTS provides the ability of inducing compression force to reduce the syndesmotic diastasis as syndesmotic bolt does. The “fiberwire and button” part fixes the distal tibiofibular joint flexibly as tightrope does. In our study, seven cases of syndesmotic diastasis were reduced using ABTS without assistance of other reduction devices, as can be achieved with syndesmotic bolt fixation [14]. ABTS provides flexible fixation of the syndesmosis and allows early weight-bearing. In all patients, full weight-bearing started at 6 weeks after fixation operation. ABTS can also maintain the reduction of the syndesmosis well. At the latest follow-up, no syndesmotic diastasis reoccurred. The mean values of MCS, TFOL and TFCS were 3.2 mm, 8.4 mm and 4.1 mm, respectively, which implied the maintenance of the anatomic reduction of distal tibiofibular joint until the latest follow-up. At the latest follow-up, all patients were satisfied with the functional outcomes according to SF-12 questionnaire. The AOFAS score was 96.3 on average, which was similar to that of patients treated with tightrope reported by Coetzee J and DeGroot H [16,21], and was higher than that of patients treated with syndesmotic bolt and standard screw fixation [6,14,17,21,25,26]. Excellent functional recoveries of the affected ankle joints were achieved in all patients, which should be mainly attribute to flexible fixation of the syndesmosis, the early weight-bearing exercise and long-term maintenance of the reduction of distal tibiofibular joint.

Several concerns should be taken into consideration during the operation. First, the device should be placed at the proper level and orientation. McBryde et al. recommended 2 cm proximal to the tibiotalar joint as the optimal level [27]. However, Miller et al. reported that the implant inserted 5 cm proximal to the tibiotalar joint could provide improved pull-out strength [28]. Although the optimal level for implant insertion is not clearly defined, we take 2–5 cm proximal to the tibiotalar joint as the proper level. In practice, the level for implant insertion is determined by referencing the position of the fibular plate or other medical hardware. In the current study, ABTS was placed parallel to the ankle joint line with a mean distance of 30.5 mm from the tibial platfond. Orientation of ABTS is also an important factor for successful fixation. In this study, the bolt was directed anteriorly at approximately 30 degrees, the same direction as recommended for tightrope technique [29]. Second, the bolt with a proper length should be selected so that the second pre-cut groove can be exposed after the nut was tightened. If the retained bolt is too long after the medial part is broken off at the second pre-cut groove, it may irritate the soft tissue, resulting in clinical symptoms. If the retained bolt is too short, over-tightening of the syndesmosis may be required to expose the second pre-cut groove. Third, routine removal of ABTS is not obligatory. ABTS can be removed in case of skin irritation, wound infection or other hardware-related complications. A recent report reviewing 11 studies with regard to syndesmotic diastasis fixed with tightrope found a 10% implant removal rate [30]. Among the patients with syndesmotic diastasis treated with a bolt, Degroot H et al reported that 5 out of 28 cases required hardware removal [14]. Most of the cases requiring hardware removal were due to prominence of implants or wound complications [14,30]. In our study, no hardware-related complications were reported. However, ABTS removal was routinely done in the current study. It is a custom or culture to remove extrinsic hardware even without symptoms in some regions and countries, such as China. In order to minimize the influence of the time of hardware removal, we removed ABTS routinely at 12-month follow-up.

This study has some limitations, including the small sample size of subjects, lack of a control group, potential influence of ankle fractures on the outcomes of the affected lower extremities, and potential bias in the collection of clinical and radiological data. Although satisfactory results were achieved in all 12 patients, a randomized controlled prospective study should be performed to compare ABTS with syndesmotic bolt and tightrope technique, which will be helpful to identify the role of ABTS in treating syndesmotic diastasis.

## Conclusions

ABTS combines the advantages of both syndesmotic bolt and tightrope techniques, which can be used to reduce the syndesmotic diastasis and simultaneously provides flexible fixation of syndesmosis. With minimally invasive and easy-to-perform technique charicteristics, ABTS seems to be an effective technique to treat syndesmotic diastasis.

## Abbreviations

ABTS: Assembled bolt-tightrope system; MCS: Medial clear space; TFCS: Tibiofibular clear space; TFOL: Tibiofibular overlap; AOFAS: American orthopedic foot and ankle society; SF-12: Short form-12; MHS: Mental health summary; PHS: Physical health summary.

## Competing interests

The authors declared that they have no competing interest.

## Authors’ contributions

YZ, GX and WC designed research; WC, GX and QZ made substantial contributions to acquiring X-ray films and doing the measurements; QZ and JW conducted the follow-ups; JW and YS analyzed data and performed statistical analysis; GX, YS and WC drafted and designed the manuscript; YZ had primary responsibility for final content; All authors read and approved the final manuscript.
